# Elevated IL-6 and IL-10 Levels as Prognostic Biomarkers in COVID-19 Pneumonia: A Comparative Study in Mexican Patients

**DOI:** 10.3390/healthcare13111245

**Published:** 2025-05-26

**Authors:** Charmina Aguirre-Alvarado, Miguel Ángel Cortes-Vázquez, Yessica Sara Pérez-González, David Eduardo Meza-Sánchez, Juan Carlos Nuñez-Enriquez, Sandra María Pinto-Cardoso, Vilma Carolina Bekker-Méndez

**Affiliations:** 1 Laboratorio de Bioquímica Farmacológica, Escuela Nacional de Ciencias Biológicas, Instituto Politécnico Nacional, Mexico City 11300, Mexico; 2 Unidad de Investigación Biomédica en Inmunología e Infectología, Hospital de Infectología “Dr Daniel Méndez Hernández”, Centro Médico Nacional (CMN) “La Raza”, Instituto Mexicano del Seguro Social (IMSS), Mexico City 02990, Mexico; 3 Hospital de Infectología “Dr Daniel Méndez Hernández”, CMN “La Raza”, IMSS, Mexico City 02990, Mexico; miguel.cortesva@imss.gob.mx (M.Á.C.-V.); draperez_medinfection@yahoo.com.mx (Y.S.P.-G.); 4 Red de Apoyo a la Investigación, Coordinación de la Investigación Científica, Instituto Nacional de Ciencias Médicas y Nutrición Salvador Zubirán, Universidad Nacional Autónoma de México, Mexico City 14080, Mexico; dmeza@cic.unam.mx; 5 Unidad de Investigación Médica en Epidemiología Clínica, UMAE Hospital de Pediatría, Centro Médico Nacional “Siglo XXI”, IMSS, Mexico City 06729, Mexico; jcarlos_nu@hotmail.com; 6 Centro de Investigación en Enfermedades Infecciosas, Instituto Nacional de Enfermedades Respiratorias Ismael Cosío Villegas, Mexico City 14080, Mexico; sandra.pintocardoso.cieni@gmail.com

**Keywords:** COVID-19, pneumonia, proinflammatory cytokines

## Abstract

Background/Objectives: Proinflammatory cytokines have been associated with poor prognosis in community-acquired and COVID-19 pneumonia. There is a paucity of reports on the cytokine release syndrome, also called cytokine storm in the Mexican population with pneumonia and COVID-19; therefore, our objective was to compare proinflammatory cytokine levels in Mexican patients without COVID-19 (non-COVID-19) and those with moderate, severe, and critical COVID-19 pneumonia. Methods: This study included 30 patients with non-COVID-19 pneumonia and 57 with COVID-19 pneumonia. Disease diagnosis and severity were determined using the radiographic pulmonary edema assessment (RALE) score. Quantification of IL-6, IL-10, and TNF-α was performed using multiplex immunoassays. A receiver operating characteristic curve was constructed to classify subjects with elevated cytokine levels. Logistic regression was used to find associations between elevated cytokine levels and the presence of COVID-19 pneumonia. Results: The severity classification of patients with COVID-19 pneumonia was as follows: moderate (n = 20), severe (n = 19), and critical (n = 18). The proinflammatory cytokines IL-6 and IL-10 were significantly increased in COVID-19 patients compared to non-COVID-19 patients (*p* < 0.005), while TNF-α levels were lower in critically ill patients with COVID-19 pneumonia. High levels of IL-6 and IL-10, adjusted for age, sex, the presence of comorbidities, and the neutrophil-to-lymphocyte ratio (NLR), showed an elevated risk (OR IL-6 = 4.02; OR IL-10 = 9.36) of presenting pneumonia and COVID-19 compared to pneumonia without COVID-19 in patients. Likewise, 61% of COVID-19 patients with elevated proinflammatory cytokines (IL-6 and IL-10) had a fatal outcome. Conclusions: Elevated levels of both IL-6 and IL-10 are a differential risk factor for developing COVID-19 pneumonia. These elevated levels were more frequently observed in Mexican COVID-19 pneumonia patients who died at the onset of the COVID-19 pandemic. It is important that they are monitored from initial diagnosis as they may be markers of a fatal outcome in severe and critical COVID-19 patients.

## 1. Introduction

Coronavirus disease 2019 (COVID-19), a highly contagious pneumonia caused by the Severe Acute Respiratory Syndrome Coronavirus 2 (SARS-CoV-2), was declared a pandemic in March 2020 by the World Health Organization (WHO) [1,2]. COVID-19 pneumonia shares etiological and clinical similarities with other respiratory syndromes caused by other coronaviruses, including Middle East Respiratory Syndrome [3], and other similar viral infectious diseases, such as influenza [4]. Quantitative reverse transcription-polymerase chain reaction (RT-PCR) testing is the gold-standard diagnostic tool for the detection of COVID-19 in respiratory samples [5]. Chest computed tomography and X-ray (CXR) plays an important role in diagnosis, classifying disease severity, guiding treatment, detecting complications, and assessing response to treatment. The quantitative radiologic score, for the radiographic assessment of lung edema (RALE), is also used to assess the severity of pulmonary edema and acute respiratory distress syndrome (ARDS) [6]. The RALE score is a reliable diagnostic tool for patients with ARDS caused by COVID-19 [7]. Depending on consolidation or ground-glass opacity, each chest radiograph is given a score between 0 and 8, ranging from the absence of any involvement (score 0) to complete lung involvement on both sides (score 8) [6,7,8]. It has been reported that unexpected respiratory distress with fatal outcomes may occur without any significant changes in pulmonary images, but only once pulmonary embolism or secondary infection is discarded. One possible reason could be lung hyperinflammation, involving the cell-mediated immunity and cytokine storms [9]. High blood levels of cytokines and chemokines have been detected in patients with COVID-19, including IL1-β, IL1RA, IL-2, IL-4, IL-6, IL-7, IL-8, IL-9, IL1-0, basic FGF2, GCSF, GMCSF, IFNγ, IP10, PDGFB, TNF-α, and VEGFA; all of these markers have been proposed as biomarkers of the severity and fatal outcome of COVID-19 [10,11,12]. Several reports have identified IL-6 as a relevant cytokine, followed by IL-10 and TNF-α, and have suggested the three cytokines to be potential therapeutic targets. It has been suggested that the cytokine storm is a direct response to pathogens, rather than as a result of specific immune pathways [13]. For example, the release of IL-6 in response to bacterial stimuli is greater than that in response to viral stimuli [14]. In contrast, elevated levels of IL-6 and IL-10 have been identified as a distinguishing feature of respiratory stress syndrome caused by adenovirus infection, as compared to cases of SARS-CoV-2 infection [15]. Therefore, describing the clinical features of COVID-19 pneumonia and quantifying cytokines at the onset of the pandemic was relevant for assessing disease severity in Mexican patients with COVID-19 pneumonia and non-COVID-19 pneumonia. Clinical laboratory markers, such as total blood count, low hemoglobin levels, polycythemia and leukocytosis with neutrophil predominance, and a decreased platelet count, have also been proposed to be indicators of disease severity, progression, and outcome, as well as high levels of liver enzyme and total bilirubin in severe and critical COVID-19 patients [16,17,18,19,20]. There is a paucity of reports on the cytokine storm in the Mexican population with pneumonia and COVID-19 [21,22]. Therefore, we compared circulating cytokine levels in Mexican patients without COVID-19 (non-COVID-19, due to unrelated causes) and with moderate, severe, and critical COVID-19 pneumonia by quantifying several proinflammatory cytokines including IL-2, IL-4, IL-6, IL-8, IL-10, TNF-α, GM-CSF, and IFN-γ. 

## 2. Materials and Methods

### 2.1. Study Population and Data Collection

Our study involved a prospective cohort of 87 adult patients (≥18 years old) with pneumonia diagnosed using CXR imaging that were admitted from April to October 2020 at “Hospital de Infectología, Centro Médico Nacional (CMN), La Raza”, “Instituto Mexicano del Seguro Social (IMSS)”, Mexico City, a referral hospital for attending COVID-19 patients.

This study was conducted in accordance with the Declaration of Helsinki and its subsequent revisions. All patients agreed to participate in this study and signed an informed consent form. This study was approved by the medical ethics and scientific committees of the “Hospital General, Gaudencio Gonzalez Garza”, CMN “La Raza”, IMSS, Mexico City, under the following protocol code: R-2020-3502-070. Exclusion criteria were age <18 years, pregnancy, or voluntary discharge. All patients who met the inclusion criteria were included. This ensured a randomized, representative sample. 

COVID-19 pneumonia patients were classified according to the clinical management of COVID-19 in accordance with the WHO [23] criteria as follows. 1. Moderate group: patients presenting with fever, respiratory symptoms, and imaging findings of pneumonia. 2. Severe group: patients with at least two of the following conditions: fever, respiratory symptoms, imaging findings of pneumonia, a respiratory frequency of 30 breaths/min, and an oxygen saturation (SpO2) of ≤90% at rest. 3. Critical group: patients with any of the following conditions: respiratory failure requiring mechanical ventilation, shock, and organ failure, needing admission into an intensive care unit. 

Demographic (age and gender), clinical (hypertension, cardiac disease, diabetes, obesity, hypothyroidism, and asthma), and analytical data (glycemia, creatinine, total bilirubin, leukocytes, lymphocytes, neutrophils, lactate dehydrogenase, aspartate amino transferase, alanine amino transferase, fibrinogen, sodium, and potassium), full blood counts, and biochemistry blood tests, parameters that were assayed by the Clinical Laboratory Service of the “Hospital de Infectología, CMN La Raza”, IMSS, were obtained from electronic medical records.

### 2.2. RALE Score

Pneumonia was diagnosed by the attending expert physician by analyzing CXR electronic images. The images were used to calculate the RALE score. These scores were obtained by dividing each lung into four segments (a total of eight segments per radiograph, Supplementary Figure S1). A score of one point was assigned to each segment with any sign of infection, such as consolidation or ground-glass opacities. Total scores represented the sum of each individual score, where the level of severity was identified according to the total points assigned to each radiograph as follows: normal—0; moderate—1–2; severe—3–6; and critical—7–8 [6]. To reduce inter-observer reliability for the RALE score, blinded repeated measurements were scored by two separate clinicians (Y.P.G and M.C.V).

### 2.3. Confirmatory Diagnosis of COVID-19

COVID-19 was confirmed by real-time reverse transcription polymerase chain reaction assay (qPCR) using throat and nasal swabs collected at admission. SARS-CoV-2 qPCR was performed using the Logix Smart™ Coronavirus 2019 (Test Kit Co-Diagnostics, Inc., Salt Lake City, UT, USA) at the “Laboratorio Central de Epidemiología”, CMN “La Raza”, IMSS, following authorization and approval from the Reference Institute Diagnosis and Epidemiological Ministry of Health, Mexico [24]. 

### 2.4. Serum Cytokine Quantification

Cytokine was carried out for all patients at admission. For the critically ill COVID-19 pneumonia patients, cytokine measurements were also carried out on days 5 and 10 after admission. A phlebotomist collected 3 ml of whole blood which was allowed to clot at room temperature for 30 min. After centrifugation for 10 min at 3000× *g*, serum was separated, aliquoted, and then frozen at −70 °C until further use. Quantification was carried out using the Bio-Plex Pro Human Cytokine 8-plex Assay version 6.1 (Catalog #M50000007A, BIO-RAD Laboratories, Milano, Italy), which included IL-2, IL-4, IL-6, IL-8, IL-10, TNF-α, GM-CSF, and IFN-γ. Quantification was performed at the “Unidad de Proteómica y Metabolómica, Instituto Nacional de Ciencias Médicas y Nutrición Salvador Zubirán”, Mexico City. 

Serum samples (50 μL) were thawed at room temperature, mixed well, and centrifuged to remove particles. Magnetic bead-based multiplex immunoassays from Bioplex-Pro human Cytokine Grp I Panel, 8-plex (Catalog # M50000007A, BIO-RAD Laboratories, Milano, Italy), were used according to the manufacturer’s instructions. 

Serum samples were used in duplicate alongside standard curves for all recombinant cytokines (range of 1–10,000 pg/mL). Data were acquired using the Bio-Plex 200 reader and analyzed using the Bio-Plex Manager software version 6.1. 

Quantifications of IL-4, GM-CSF, and IFN-γ were below the standard curve and were not included in our analysis.

### 2.5. Statistical Analysis

Categorical variables were expressed as frequencies and percentages, and continuous variables were expressed as medians and interquartile ranges or means ± standard deviations according to their distribution. Normal distribution was tested using Kolmogorov–Smirnov normality test. ANOVA or Kruskal–Wallis tests with post hoc Tukey or Dunn multiple tests for adjusting for multiple comparisons were used for group comparisons when dependent variables were continuous and according to their distribution. Chi-square testing was used for categorical variables. A *p* value of <0.05 indicated statistical significance. An ROC curve was used to determine the best cytokine concentration thresholds and classify subjects accordingly (with high cytokine values). Binary logistic regression was used to assess associations between our outcomes: diagnosis of COVID-19 and predictor variables and high cytokine levels. We used a Venn diagram to represent the frequencies of single relevant cytokines and combined relevant cytokines; these were compared using the Chi-square test. The Kaplan–Meier method and the predicted survivor function of a Cox proportional hazard model were used to estimate the survival probability of severe and critical COVID-19 patients as deaths only occurred in these groups. The log-rank test was used to assess survival equality. All variables had missingness of less than 5%; missing values were imputed with the median value. The SPSS version 25.0 statistical software was used for statistical analysis, and figures were plotted using GraphPad 6.0 and R (ggVennDiagram and survival packages).

## 3. Results

### 3.1. Demographic and Clinical Characteristics and Laboratory Findings of Patients at Admission

Demographic and clinical characteristics are summarized in Table 1. This study included 87 patients with pneumonia: 30 (35%) tested negative for COVID-19 (non-COVID-19) and 57 (65%) tested positive for COVID-19.

Using the WHO criteria and supported by the RALE score (Figure S1), we stratified all COVID-19 patients based on the severity of COVID-19 as follows: 20 (35%) moderate, 19 (33%) severe, and 18 (32%) critical. Severe and critically ill COVID-19 patients were hospitalized. Our cohort included 50 males (56%) and 37 females (44%), with a median age of 49 years. No statistical difference was observed between groups in terms of sex at birth and age (*p* = 0.29 and *p* = 0.27, respectively). The most frequent comorbidity was hypertension, with 10 cases (32%) in non-COVID-19, 8 (42%) in moderate COVID-19, 8 (42%) in severe COVID-19, and 8 (44%) in critical COVID-19 pneumonia patients, followed by diabetes and obesity. No statistical differences were observed between groups for hypertension and diabetes (*p* = 1.0 and *p* = 0.97, respectively). Fatal outcomes were observed in 10 (60%) severe COVID-19 patients and in 13 (72%) critical COVID-19 patients. None of the non-COVID-19 or moderate COVID-19 patients had a fatal outcome.

Table 2 shows data on the complete blood work at the patients’ admission. We found significantly higher levels of leucocytes and neutrophils and a high neutrophil-to-lymphocyte ratio (NLR) (*p* = 0.018, *p* = 0.002, and *p* = 0.005, respectively) in critical COVID-19 pneumonia patients compared to those in non-COVID-19 pneumonia patients. The reference normal range was 4500–11000 cells/μL, 1500–8000 cells/µL, and 0.78–3.53 for leucocytes, neutrophils, and the NLR, respectively. In addition, serum albumin concentration (2.93 ± 0.4 g/dL) was significantly lower in the critical group compared to the non-COVID-19 group, and this concentration was below the reference normal range (3.5 to 5.5 g/dL). 

### 3.2. Inflammatory Cytokine Profile in Patients with Pneumonia at Admission

**Figure 1:**
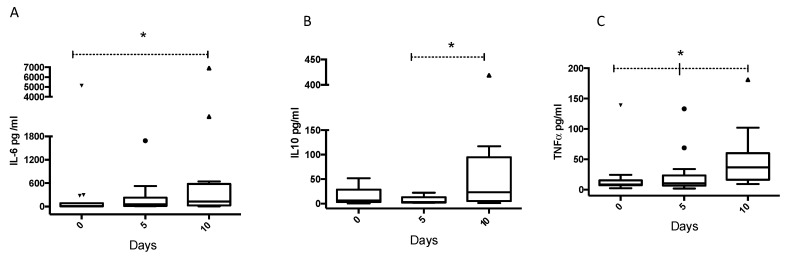
Cytokine levels in critical COVID-19 patients. (**A**) IL-6, (**B**) IL-10, and (**C**) TNF-α levels were measured in the serum collected from critical COVID-19 patients on days 0, 5, and 10. * *p* < 0.05. *p* values were calculated using Kruskal–Wallis testing with post hoc Dunn′s multiple test.

Cytokine profiles are shown in Table 3. Circulating levels of IL-10, IL-6, and TNF-α were significantly different between groups (P_IL-10_ < 0.001; P_IL-6_ = 0.0307; P_TNF-α_ = 0.0034). Moderate COVID-19 pneumonia patients had higher levels of IL-6 compared to non-COVID-19 patients. Moderate and severe COVID-19 patients but not critical COVID-19 patients showed higher levels of IL-10 compared to non-COVID-19 patients. TNF-α concentrations were lower in critical COVID-19 patients compared to those in moderate and severe COVID-19 patients. Serum IL-2 and IL-8 levels were not significantly different. Circulating levels of IL-10, IL-6, and TNF-α in critical COVID-19 patients were further assessed on days 5 and 10 (Figure 1). We observed a significant increase in IL-6, IL-10, and TNF-α levels (*p* < 0.05), with the highest levels observed at day 10, suggesting a continuous increase from day 0, in particular for IL-6 and TNF-α.

### 3.3. Associations Between COVID-19 Pneumonia and Circulating Cytokines Using Logistic Regression

To stratify patients with high levels of circulating cytokines, we used the AUC with the best specificity and sensitivity (Supplementary Figure S2). The AUCs were 0.74 (95% CI = 0.75–0.774), 0.62 (95% CI = 0.607–0.645), and 0.52 (95% CI = 0.806–0.375) for IL-10, IL-6, and TNF-α, corresponding to 3, 16, and 11.3 pg/ml, respectively. 

A binary logistic regression analysis model showed that pneumonia caused by COVID-19 was associated with levels of IL-6 > 16 pg/ml (OR = 4.02; *p* < 0.01; 95% CI = 1.43–11.34) and IL-10 > 3 pg/ml (OR = 9.36; *p* < 0.01; 95% CI = 3.21–21.44). This model was adjusted for age, sex, comorbidities, and the NLR. High levels of TNF-α were not associated with COVID-19 pneumonia (Table 4).

**Figure 2:**
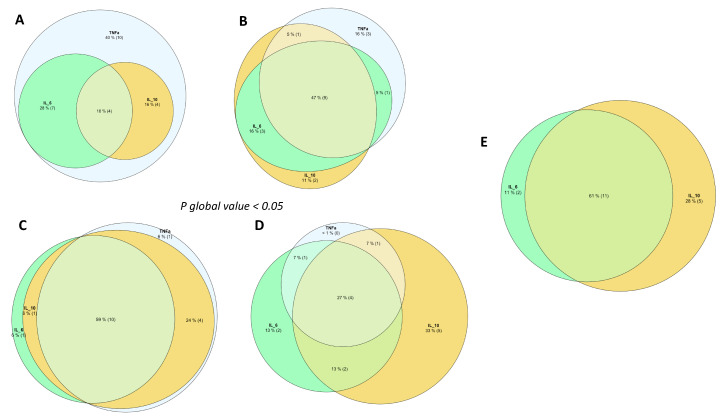
Venn diagrams representing the overlaps of the number of patients with above-threshold cytokine levels (IL-6 = 16.0 pg/mL; IL-10= 3.0 pg/mL; TNF-α = 11.3 pg/mL) in (**A**) non-, (**B**) moderate, (**C**) severe, (**D**) critical, and (**E**) deceased COVID-19 patients. (**A**–**D**) Frequencies were compared using the Chi-square test. None of the COVID-19 patients who died presented above-threshold TNF-α levels.

When comparing the frequency of patients with elevated levels of IL-10, IL-6, and TNF-α and their combinations or overlaps, we found a significant difference (*p* < 0.001) between the non-, moderate, severe, and critical COVID-19 groups (Figure 2A–D). Moderate and severe COVID-19 patients showed the highest percentage of overlap with high levels of IL-10 and IL-6 and TNF-α (47 % and 58%, respectively). In patients who had a fatal outcome, 61% had a combination of high IL-6 and IL-10 levels and none had high TNF-α levels (Figure 2E). The Kaplan–Meier survival curves were graphed only for patients in the severe and critical COVID-19 groups, and they did not show a statistically significant difference between the high and low levels of IL-6, IL-10, and TNF-α at admission (Supplementary Figure S3). The median (IQR) survival of patients with pneumonia and COVID-19 with a fatal outcome was 11 (4.5–16) days. Furthermore, the median survival of patients with pneumonia and COVID-19 was 11 (4–12), 9 (2–11.25), and 11 (5.5–13) days for patients with high levels of IL-6, IL-10, and TNF-α, respectively.

The results were adjusted by age, sex, the presence of comorbidities (yes/no), and the NLR. The results are from separate analyses because a high correlation among cytokines was identified (>0.30).

## 4. Discussion

The most frequent etiological agent causing the hospitalization of community-acquired pneumonia patients in Latin America is Streptococcus pneumoniae [25]. Bacterial pneumonia has different treatment modalities and prognoses when compared with COVID-19 [4]; therefore, differential diagnosis is of great importance. Although the diagnosis of SARS-CoV-2 infection is performed at the molecular level, medical imaging techniques play an important role in supporting diagnosis and classifying the disease. In this sense, CXR, whether conducted in conventional X-ray rooms or with portable units, is the first-line imaging test because it is widely available and inexpensive. The quantitative radiological RALE score is an excellent tool to evaluate the severity of pulmonary infiltrates based on CXR images, and it is increasingly used in critically ill, invasively ventilated patients when no tomography is available [7,8]. According to our study, the evaluation of CXR images using the RALE score supported by clinical data resulted in a useful tool to classify the severity of COVID-19 pneumonia in patients.

On the other hand, clinical, laboratory, and inflammatory cytokine profiles are useful data that should be considered and included when a new infectious disease emerges. In this context, the clinical description of COVID-19 pneumonia and the associated cytokine profile in a Mexican population at the onset of the pandemic might be useful to aid in stratifying disease severity. In our study, we described the demographical and clinical characteristics of patients with COVID-19 stratified by severity and assessed associations to infer the potential use of inflammatory cytokines (IL-2, IL-4, IL-6, IL-8, IL-10, TNF-α, GM-CSF, and IFN-γ) as biomarkers of COVID-19 pneumonia. We found that COVID-19 pneumonia patients were predominantly male. Our data are consistent with a recent meta-analysis of 3 million patients, in which being male was a risk factor for intensive care unit admission and death [26]. The three main comorbidities observed in our study were hypertension, diabetes, and obesity, which are consistent in worldwide reports [27] and in the Mexican population [28].

Critical COVID-19 patients showed a significant increase in the neutrophil count and NLR upon admission. Several studies have reported a high NLR to be a critical early warning sign in COVID-19 pneumonia patients [17], the NLR has been suggested as a differentiating factor for the diagnosis of viral and bacterial pneumonia [29]. In this cohort, we did not find a significant difference in various components including creatinine, fibrinogen, and liver enzyme activities at admission, although several studies have suggested that these might be early significant biomarkers of kidney and liver injury associated with poor outcomes in COVID-19 patients [30]. 

There is a substantial amount of data on cytokine storm syndrome in COVID-19 patients, particularly high circulating levels of IL-6, IL-10, IL-2R, IL-8, and TNF-α in COVID-19 patients with high mortality rates [31,32]. Interestingly, levels of these circulating cytokines are similar to those reported in any local inflammatory response following community-acquired pneumonia [33]. Therefore, it is relevant to quantify cytokine levels in Mexican patients who develop pneumonia with and without COVID-19.

In this study, we observed that the circulating levels of IL-6, IL-10, and TNF-α were significantly different between COVID-19 pneumonia and non-COVID-19 pneumonia patients at admission; both IL-6 and IL-10 levels were found to be increased in the critical COVID-19 group while the TNF-α levels was found to be decreased. The largest significant difference was observed for IL-10 when comparing non-COVID-19 patients to COVID-19 patients with moderate and severe symptoms. We suggest that IL-10 might exert a potent immunomodulatory effect. IL-10 is also an antifibrotic agent for pulmonary fibrosis caused by viruses [34]. Some studies have reported lower levels of IL-10 in critical-ill COVID-19 patients [35]. In addition, the pathogen-triggered release of IL-10 levels is associated with bacterial persistence in the lungs through neutrophil infiltration [36]. This may suggest that IL-10 is a distinctive biomarker between critical COVID-19 pneumonia and bacterial pneumonia.

In most studies conducted in Asia and Europe, circulating IL-6 levels correlate with COVID-19 severity. In our cohort, we found a significant increase in IL-6 at admission in moderate COVID-19 patients. In a similar study on Mexican COVID-19 patients during the early pandemic, the authors reported that IL-6 levels were significantly higher in the non-survivor group when compared with those in the survivors [37]. This study had a limited number of participants (n = 38). Collectively, these data warrant further studies on the Mexican population to better understand pneumonia caused by COVID-19. Other studies report higher levels of TNF-α in patients with severe or critical COVID-19 [38,39]. Yet, our data showed lower levels of TNF-α in critical COVID-19 patients at admission. It is important to note that we measured TNF-α levels at admission, and then at day 5 and day 10, and they showed an increase at day 10 in critical COVID-19 patients. The discrepancy between our data and other reports might be due to the fact that most cross-sectional reports do not specify when measurements were taken. Conversely, longitudinal reports support the idea that TNF-α levels increase until day 14, followed by a decrease [40]. This highlights the importance of dynamic studies that can provide more reliable data on the concentration of cytokines according to disease severity. Similar results were reported by Huang and colleagues, who reported increased circulating levels of IL-6 and IL-10 in critical COVID-19 patients. They did not report any differences in IL-2, IL-4, and IFN-γ levels [9]. We also found no differences for IL-2 and IL-8 (Table 3). Collectively, these findings might suggest that IL-6 and IL-10 are differential biomarkers of critical COVID-19 pneumonia and non-COVID-19 pneumonia. Our results also showed an increase in circulating IL-6, IL-10, and TNF-α levels in critical COVID-19 patients after 10 days of hospitalization, highlighting the importance of quantifying these cytokines longitudinally in COVID-19 patients. 

High levels of IL-6 and IL-10 were associated with COVID-19 pneumonia patients after adjusting for age, sex, the presence of comorbidities, and the NLR. We suggest that both IL-6 and IL-10 might be useful biomarkers to improve diagnosis and severity stratification in COVID-19 patients and possibly tailor treatment accordingly. Our data are concordant with other publications that suggest the relevance and importance of monitoring circulating levels of IL-6 and IL-10 as useful predictors of early diagnosis for severe COVID-19 patients [22,41,42]. Both pleiotropic cytokines IL-6 and IL-10 are produced by cells including macrophages, lymphocytes, endothelial cells, epithelial cells, and fibroblasts at sites of inflammation [43]. High levels of IL-6 and IL-10 could be related to the overactivation of Th2 immune-mediated responses, resulting in disease progression to the critical COVID-19 stage or even death [44]. High levels of IL-6 and IL-10 could be useful biomarkers to discriminate non-COVID-19 pneumonia from COVID-19 pneumonia, which is in line with other reports where the IL-6 and IL-10 ratio has been suggested as a useful biomarker to discriminate severe pneumonia cases at admission [45]. In our study, TNFα was not associated with the diagnosis of COVID-19 pneumonia, although several authors have pointed out that TNF-α is one of the most important cytokines present during the cytokine storm and is associated with disease severity [46]. In this study, high levels of TNF-α were more frequent in non-COVID-19 pneumonia patients. Previous reports on pneumonia patients showed that high serum levels of inflammatory cytokines, such as TNF-α, have been correlated with worse outcomes in community-acquired pneumonia patients [47]. Notably, none of the COVID-19 patients who died presented high levels of TNF-α. Although our results did not show an association between survival and high levels of IL-6, IL-10, and TNF-α, the survival curves of severe COVID-19 patients were similar to those of critical COVID-19 patients in regard to high IL-10 levels; elevated IL-10 levels could be an early warning sign of low survival in patients with pneumonia and COVID-19. Similar results were reported by Jafrin and colleagues. They found that the mean concentration of circulating IL-10 was higher in deceased COVID-19 patients compared to those in COVID-19 patients who survived [19]. In another study by del Valle and colleagues, they reported that high levels of IL-6 and TNF-α were associated with low survival in patients with COVID-19 [48]. They did not stratify patients by COVID-19 severity, so the non-survivor group was composed of severe and critical patients. In addition, their cut-off points for determining high levels of IL-6 and TNF-α were different. The latter two might explain discrepancies between our studies. 

Our results show that combined high levels of IL-6 and IL-10 in patients with pneumonia can have a significant impact on clinical practice by differentiating between COVID-19 and non-COVID-19 pneumonia. This might be useful in aiding in choosing which pharmacological treatment a patient should receive, thus enabling personalized medicine. We suggest that measuring circulating levels of IL-6 and IL-10 should be implemented and routinely quantified for patients with pneumonia and suspected COVID-19.

## 5. Conclusions

Our study was performed during the early COVID-19 pandemic (2020) and reports clinical and epidemiological characteristics of patients with and without COVID-19 and pneumonia during period of time when there was considerable limitations on COVID-19 treatment. We suggest that monitoring cytokine storm syndrome during diagnosis in different COVID-19 patient groups is warranted. It might provide relevant data on how useful these biomarkers might be in discriminating between COVID-19 pneumonia in critical patients and non-COVID-19 pneumonia. High levels of both IL-6 and IL-10 were found in critically ill COVID-19 patients, which were at the highest risk of death. In conclusion, our results highlight the importance of assessing both IL-6 and IL-10 as possible biomarkers for COVID-19 pneumonia in the Mexican population. Yet, further work is warranted including larger cohorts. 

## 6. Limitations

We acknowledge that the present study has limitations. Firstly, we included a small number of patients, in contrast with studies conducted in Asia and Europe. Yet, the data presented in this study were collected at the onset of the pandemic in Mexico (2020). The subjective nature of interpreting the extent and density of opacities in chest radiographs must be acknowledged. Yet, we mitigated this limitation by having two expert physicians generate individual reports and a consensus report, therefore reducing subjectivity. Third, cytokine production is highly variable and depends on genetic and environmental factors that were not accounted for in this study. The scarcity of reports on the cytokine storm in patients with COVID-19 in the Mexican population makes it difficult to compare our data. As we used a multiplex assay, some cytokines including IL-4, GM-CSF, and IFN-γ could not be reliably quantified and were therefore excluded from our study. This is often an issue due to the nature of the assay. In such cases, it might be preferable to use individual assays for each cytokine. Finally, the cross-sectional nature of our study did not allow for causality to be established; we were only able to report associations. Our study also has strength; as mentioned previously, the data presented in this study were collected at the onset of the pandemic in Mexico (2020), we stratified the cohort according to disease severity for COVID-19 patients, and we assessed circulating cytokine levels longitudinally for critically ill COVID-19 patients. 

## Figures and Tables

**Table 1:** Clinical and demographical characteristics of our study cohort.

	Non-COVID-19 (n = 30)	COVID-19 (n = 57)			
		Moderate (n = 20)	Severe (n = 19)	Critical (n = 18)	*p*
Gender (female/male)	16/15	5/14	9/10	7/11	0.29
Age, years (mean ± SD)	55 (40.5–66.5)	46 (39.5–61.0)	48 (34.5–61.5)	51.50 (41.25–60.0)	0.27
Comorbidities, n (%)					
Hypertension	10 (32%)	8 (42%)	8 (42%)	8 (44%)	1.0
Diabetes	5 (16%)	4 (21%)	4 (21%)	4 (22%)	0.97
Obesity	2 (2%)	1 (5%)	1 (5%)	1 (5%)	NA
HIV	---	1 (5%)	1 (5%)	0	NA
Hypothyroidism	1 (3%)	2 (10%)	0	0	NA
Asthma	0	0	1 (5%)	0	NA
Cardiac Disease	0	1 (5%)	0	0	NA
Death	0	0	10 (60 %)	13 (72%)	NA

**Table 2:** Complete blood work of non-COVID-19 and COVID-19 patients stratified by disease severity.

	Non-COVID-19 (n = 30)	COVID-19					
		Moderate (n = 20)	Severe (n = 19)	Critical (n = 18)	*p* Value	Group 1 vs. Group 2	*p*.adjust
Hb (g/dL)	14.44 ± 2.39	14.5 ± 2.38	14.1 ± 1.64	13.5 ± 1.93	ns		
Leucocytes (10^3^ cells/µL)	7.3 (5.9–11.5)	7.8 (6.58–13.1)	7.7 (6.45–8.85)	12.7 (9.2–14.6)	0.002 *	N vs. C	0.018
Neutrophils (10^3^ cells/µL)	5.4 (3.9–8.9)	6.83 (5.39–11.4)	6.0 (4.58–6.4)	10.9 (7.85–13)	0.001 *	N vs. C	0.002
Lymphocytes (10^3^ cells/µL)	1.02 (0.68–1.43)	0.92 (0.62–1.16)	1.04 (0.71–1.33)	0.59 (0.42–1.01)	ns		
N/L ratio	4.97 (2.32–11.85)	9.71 (3.72–13.61)	5.02 (3.6–8.97)	15.41 (9.93–50.41)	0.003 *	N vs. C	0.005
Glucose (mg/dL)	106 (90–170)	115 (102–152)	114 (97–136)	149 (112–190)	ns		
Creatinine (mg/dL)	0.93 (0.72–1.14)	0.96 (0.86–1.37)	1.06 (0.8–1.52)	1.13 (0.8–1.91)	ns		
Albumin (g/dL)	3.39 ± 0.56	3.76 ± 0.523	3.64 ± 0.424	2.93 ± 0.4	<0.001 *	N vs. C M vs. C S vs. C	<0.001 <0.001 <0.001
LDH (U/L)	536 (270.5–669)	524 (373–684)	592 (404–961)	539 (420–780)	ns		
AST (U/L)	50 (19.5–64.25)	45 (34.8–52.3)	57.5 (33.5–93)	90.1 (69.5–291)	ns		
ALT (U/L)	38 (24.2–68)	37 (25.8–84.8)	49.5 (41.5–72)	42.5 (26.3–56.8)	ns		
Fibrinogen (mg/dL)	874 ± 362.4	928 ± 240	939 ± 285	760 ± 401	ns		
Sodium (mmol/L)	137 (136–148.8)	137 (135–141)	136 (131–140)	141 (139–146)	ns		
Potassium (mmol/L)	3.95 (3.69–4.3)	4.0 (3.85–4-69)	4.15 (3.79–4.58)	4.62 (4.03–4.91)	ns		

Data are presented as means ± standard deviations or medians (IQR). *p* values were calculated using ANOVA or Kruskal–Wallis test, as appropriate. *p*.adjust: *p* values were adjusted using post hoc Tukey or Dunn tests. * *p* < 0.05. Abbreviations: ALT, alanine aminotransferase; AST, aspartate aminotransferase; IQR, interquartile range; LDH, lactate dehydrogenase; N, non-COVID; M, moderate; N/L: neutrophil-to-lymphocyte ratio, S, severe; C, critical; ns, non-significant.

**Table 3:** Comparisons of circulating cytokine levels between non-COVID-19 and COVID-19 patients at admission.

Cytokine (pg/mL)	Non-COVID-19 (n = 30)	COVID-19					
		Moderate (n = 20)	Severe (n = 19)	Critical (n = 18)	*p* Value	Group 1 vs. Group 2	*p*.adjust
IL-6	8.83 (3.27–23.23)	47.64 (14.59–71.83)	16.55 (4.64–64.88)	19.43 (6.09–237.2)	0.03 *	N vs. M	0.034
IL-10	1.260 (0.72–2.9)	12.72 (4.04–19.8)	23.75 (8.4–32.03)	5.92 (3.05–26.29)	<0.001 *	N vs. M N vs. S	0.005 0.002
TNF-α	17.87 (11.87–26.86)	13.58 (5.09–27.71)	21.12 (14.2–21.17)	8.93 (7.59–16.38)	0.003 *	M vs. C S vs. C	0.008 0.008
IL-2	1.1 (0.77–1.34)	1.27 (1.03–2.75)	1.10 (0.66–1.6)	0.92 (0.53–1.45)	ns		
IL-8	17.43 (8.13–52.9)	14.99 (7.99–19.57)	6.22 (4.2–21.4)	12.88 (8.4–49.37)	ns		

Values are expressed as pg/mL and median (IQR). *p* values were obtained using Kruskal–Wallis test. *p*.adjust: *p* values were adjusted using post hoc Dunn test. * *p* < 0.05. Abbreviations: N, non-COVID; M, moderate; S, severe; C, critical; ns, non-significant.

**Table 4:** Logistic regression analyses comparing high levels of cytokines and pneumonia caused by COVID-19.

Cytokine Level	Non-COVID-19 (n)	COVID-19 (n)	OR	95% Confidence Interval	*p*
IL-6 (>16 pg/mL)	11	34	4.02	1.43–11.34	<0.01
IL-10 (>3 pg/mL)	8	42	9.36	3.21–27.44	<0.01
TNF-α (>11.3 pg/mL)	25	35	2.91	0.94–9.02	0.06

## Data Availability

The datasets used and/or analyzed during the current study are available from the corresponding author upon reasonable request.

## Supplementary Materials

The following supporting information can be downloaded at https://www.mdpi.com/article/10.3390/healthcare13111245/s1. Figure S1: RALE score determination; Figure S2: Serum cytokine ROC curves for threshold determination; Figure S3. Kaplan–Meier plots comparing high and low levels of cytokines.

## Abbreviations

The following abbreviations are used in this manuscript:

ALT	alanine aminotransferase
AST	aspartate aminotransferase
COVID-19	coronavirus disease 2019
CXR	chest X-Ray
CMN	National Medical Center
GM-CSF	granulocyte–macrophage colony-stimulating factor
IL	interleukin
IMSS	Mexican Institute of Social Security
INF-γ	interferon gamma
IQR	interquartile range
LDH	lactate dehydrogenase
NLR	neutrophil/lymphocyte ratio
OR	odds ratio
qPCR	real-time reverse transcription polymerase chain reaction
RALE	radiographic pulmonary edema assessment
ROC	receiver operating characteristic
TNF-α	tumor necrosis factor
