# Supplementary material for: Elevated IL-6 and IL-10 Levels as Prognostic Biomarkers in COVID-19 Pneumonia: A Comparative Study in Mexican Patients
Source: Healthcare (Basel). 2025 May 26;13(11):1245. doi: 10.3390/healthcare13111245 (PMC12155521; doi:10.3390/healthcare13111245)
Supplement: Supplementary file 1 [file healthcare-13-01245-s001.zip › figure S1.pdf]

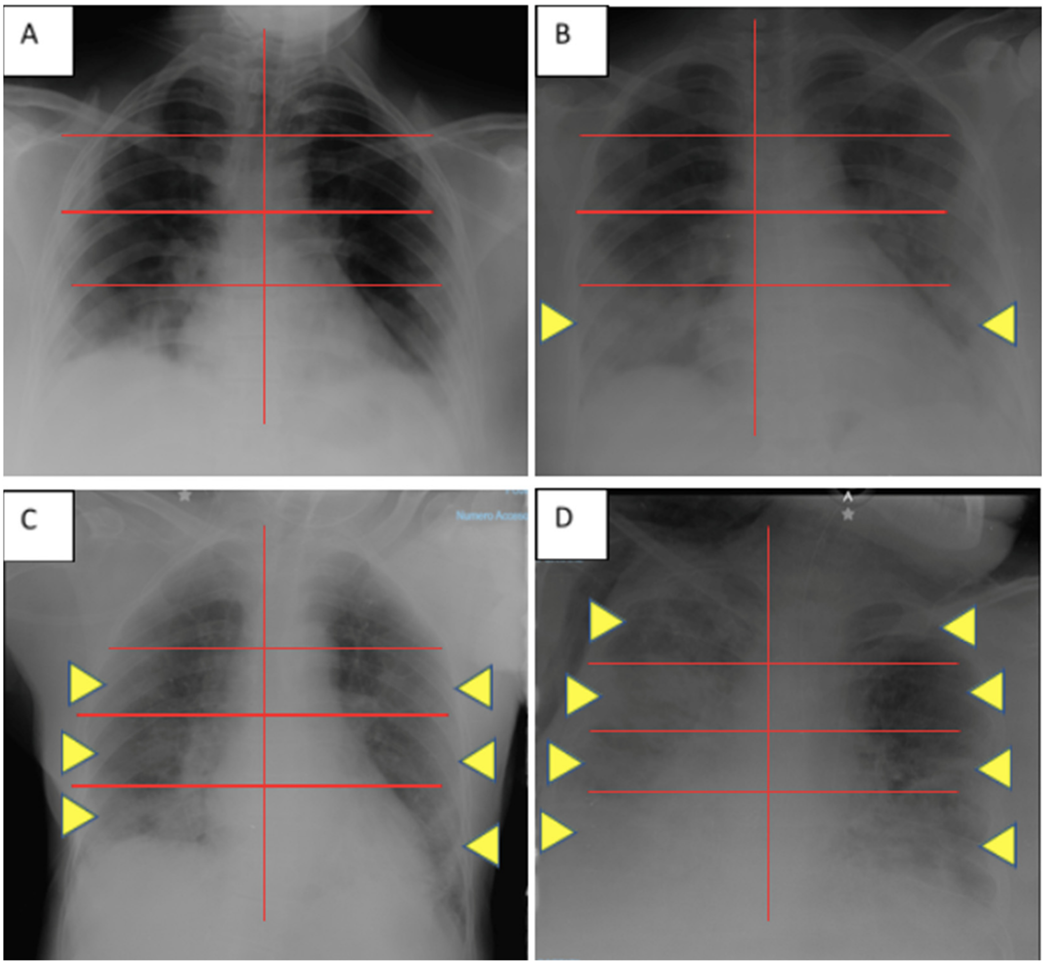

Figure S1. RALE score determination. Anteroposterior chest radiographic representative images of the studied population. Each segment is assigned a RALE score if there is any sign of infection (yelow arrows). A) image of hospitalized Non-COVID patient with diagnosis ruled out for COVID-19 with negative PCR for SARS-CoV-2;(B-D) image of hospitalized patients with positive PCR for SARS-CoV-2 divided into eight segments. B) the image show a ground glass in the lower lung area, affecting 2 segments, which according to the RALE score corresponds to mild involvement; C) showing ground glass in the middle and lower lung area, affecting 6 segments, which to moderate involvement; D) image showing ground glass in the upper, middle and lower lung area, affecting 8 segments, which corresponds to severe involvement.
