# Supplementary material for: Elevated IL-6 and IL-10 Levels as Prognostic Biomarkers in COVID-19 Pneumonia: A Comparative Study in Mexican Patients
Source: Healthcare (Basel). 2025 May 26;13(11):1245. doi: 10.3390/healthcare13111245 (PMC12155521; doi:10.3390/healthcare13111245)
Supplement: Supplementary file 1 [file healthcare-13-01245-s001.zip › figure S3.pdf]

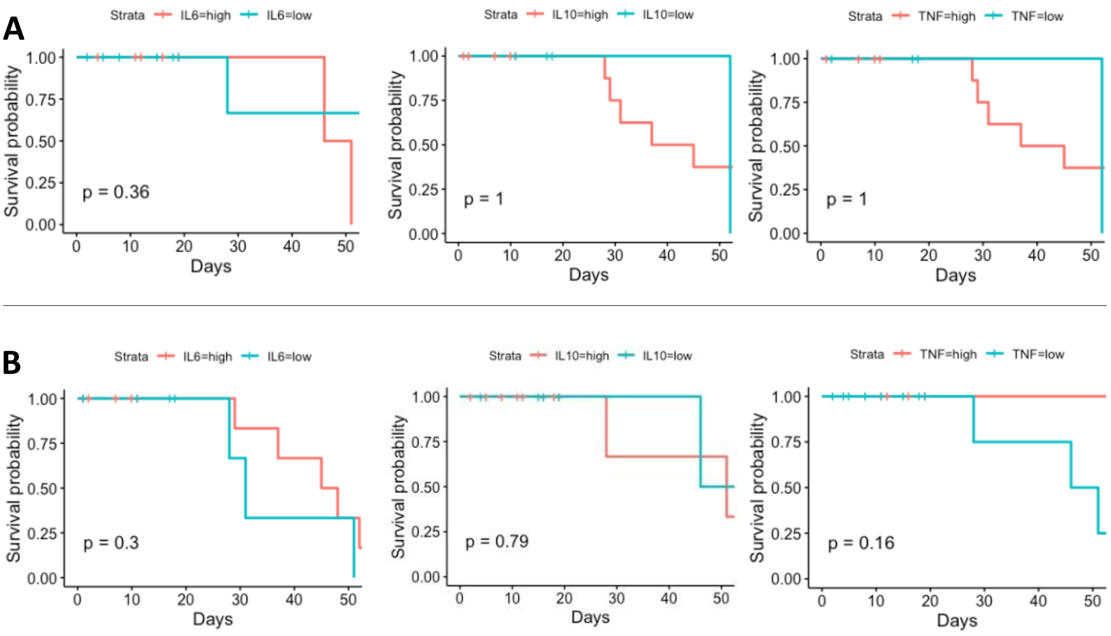


**Figure S3**. Kaplan-Meier plots comparing high and low levels of cytokines. IL-6, IL-10 and TNF◎ mortality between severe (A) and critical (B) COVID-19 patients.
