# Supplementary figures and images for: Elevated IL-6 and IL-10 Levels as Prognostic Biomarkers in COVID-19 Pneumonia: A Comparative Study in Mexican Patients
Source: Healthcare (Basel). 2025 May 26;13(11):1245. doi: 10.3390/healthcare13111245 (PMC12155521; doi:10.3390/healthcare13111245)

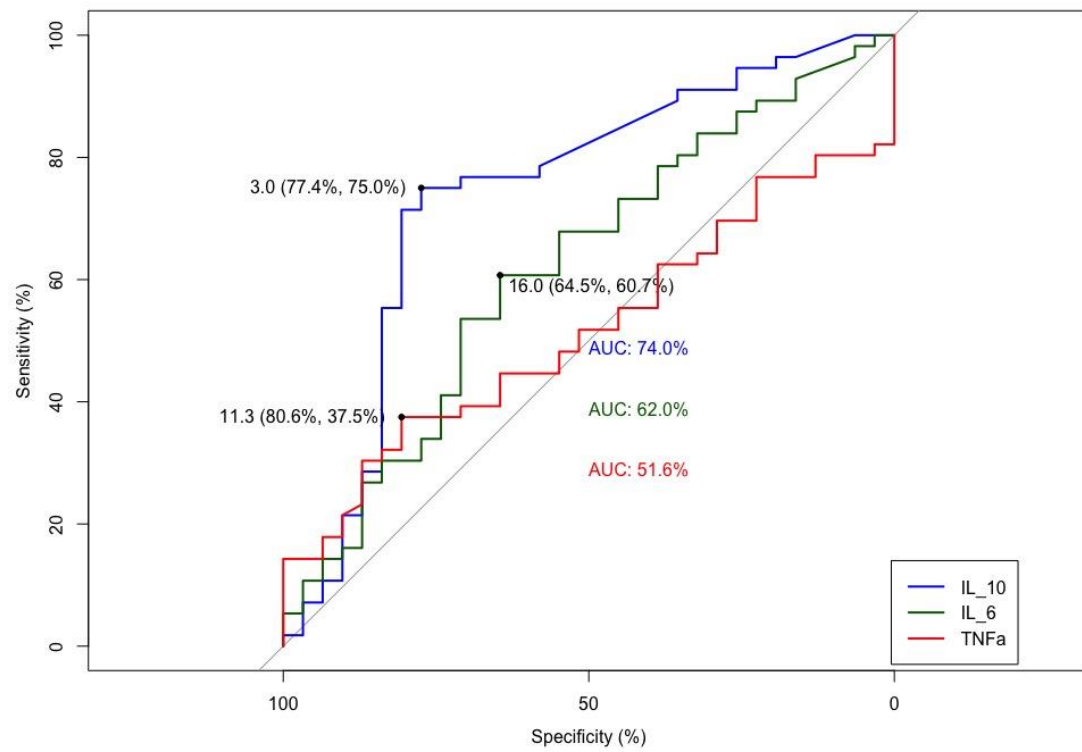

**Figure S2:** Serum cytokines ROC curves for threshold determination. IL-10 (blue), IL-6 (green) and TNFα (red).

Supplement: Supplementary file 1 [file healthcare-13-01245-s001.zip › figure S2.pdf]
